# Gynecology Meets Big Data in the Disruptive Innovation Medical Era: State-of-Art and Future Prospects

**DOI:** 10.3390/ijerph18105058

**Published:** 2021-05-11

**Authors:** Rola Khamisy-Farah, Leonardo B. Furstenau, Jude Dzevela Kong, Jianhong Wu, Nicola Luigi Bragazzi

**Affiliations:** 1 Clalit Health Service, Akko, Azrieli Faculty of Medicine, Bar-Ilan University, Safed 13100, Israel; rkhamisy@yahoo.com; 2 Department of Industrial Engineering, Federal University of Rio Grande do Sul, Porto Alegre 90035-190, Brazil; leonardo.furstenau@ufrgs.br; 3 Laboratory for Industrial and Applied Mathematics (LIAM), Department of Mathematics and Statistics, York University, Toronto, ON M3J 1P3, Canada; jdkong@yorku.ca (J.D.K.); wujhhida@gmail.com (J.W.)

**Keywords:** big data, fast and smart data, disruptive innovation medical era, gynecology

## Abstract

Tremendous scientific and technological achievements have been revolutionizing the current medical era, changing the way in which physicians practice their profession and deliver healthcare provisions. This is due to the convergence of various advancements related to digitalization and the use of information and communication technologies (ICTs)—ranging from the internet of things (IoT) and the internet of medical things (IoMT) to the fields of robotics, virtual and augmented reality, and massively parallel and cloud computing. Further progress has been made in the fields of addictive manufacturing and three-dimensional (3D) printing, sophisticated statistical tools such as big data visualization and analytics (BDVA) and artificial intelligence (AI), the use of mobile and smartphone applications (apps), remote monitoring and wearable sensors, and e-learning, among others. Within this new conceptual framework, big data represents a massive set of data characterized by different properties and features. These can be categorized both from a quantitative and qualitative standpoint, and include data generated from wet-lab and microarrays (molecular big data), databases and registries (clinical/computational big data), imaging techniques (such as radiomics, imaging big data) and web searches (the so-called infodemiology, digital big data). The present review aims to show how big and smart data can revolutionize gynecology by shedding light on female reproductive health, both in terms of physiology and pathophysiology. More specifically, they appear to have potential uses in the field of gynecology to increase its accuracy and precision, stratify patients, provide opportunities for personalized treatment options rather than delivering a package of “one-size-fits-it-all” healthcare management provisions, and enhance its effectiveness at each stage (health promotion, prevention, diagnosis, prognosis, and therapeutics).

## 1. Theoretical Background: The Current Medical Era as the “Disruptive Innovation Era”

Tremendous scientific and technological achievements have been revolutionizing the current medical era, shaping the way in which physicians practice their profession and deliver healthcare provisions. This is due to the convergence of various advancements related to digitalization and the use of information and communication technologies (ICTs)—ranging from the internet of things (IoT) and the internet of medical things (IoMT) to the fields of robotics, virtual and augmented reality, and massively parallel and cloud computing. Further progress has been made in the fields of addictive manufacturing and three-dimensional (3D) printing, sophisticated statistical tools such as big data visualization and analytics (BDVA) and artificial intelligence (AI), the use of mobile and smartphone applications (apps), remote monitoring and wearable sensors, and e-learning, among others.

Other drivers of and catalysts for innovation are represented by developments in the fields of P6 medicine (where the 6 Ps stand for personalized, predictive, preventive, participatory, psycho-cognitive, and public), nanomedicine, molecular medicine, and nanotherapy—including the use of new nano(bio)technological materials, and genetic, genomic, and post-genomic engineering and editing [1].

**Figure 1:**
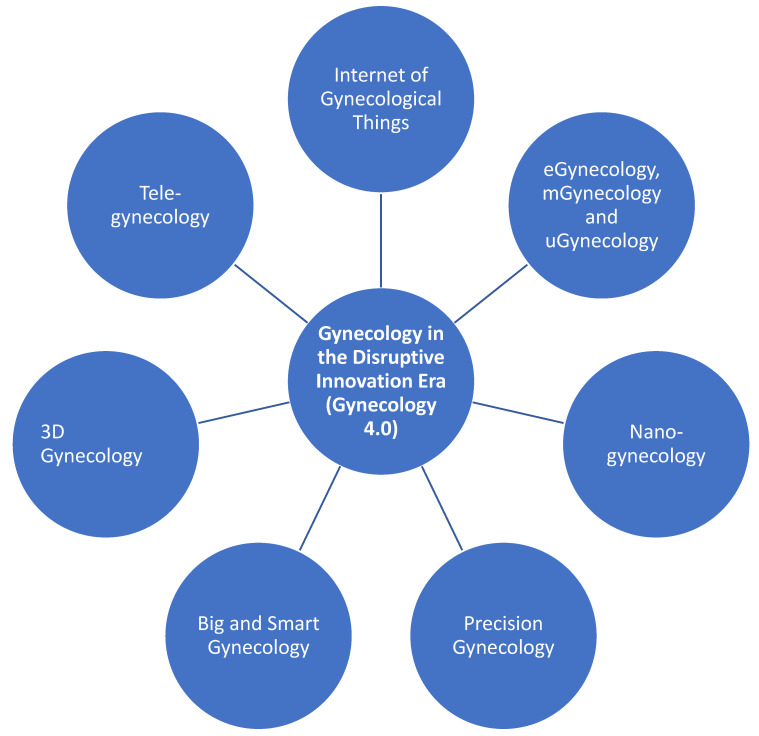
The convergence of advancements and progress from different fields (information and communication technologies, digitalization, molecular biology, biochemistry, biophysics, nano-engineering, and nano(bio)technology).

All these profound changes characterize the so-called “disruptive era” (Figure 1) [2], which is dramatically impacting all medical specializations—including gynecology. As such, gynecology has recently branched out into various sub-specialties, like:

- the internet of gynecological things.
- eGynecology (from the combination of gynecology and eHealth, which, in turn, is an abbreviation for electronic health, conceived as the use of electronic devices and tools aimed at improving and enhancing health-related outcomes) [3].
- mGynecology (from the combination of gynecology and mHealth, which, in turn, is an abbreviation for mobile health, that is to say the use of mobile and wireless technologies to achieve health-related purposes and objectives).
- uGynecology (from the combination of gynecology and uHealth, which, in turn, is an abbreviation for ubiquitous health, or the application of wearable sensors to constantly monitor in real-time the health status of individuals).
- tele-gynecology (from the combination of gynecology and tele-health/tele-medicine, which refers to the innovative and emerging practice of delivering healthcare provisions remotely).
- nano-gynecology (from the combination of gynecology and nano-medicine, which is the practice of the medicine at the nano-scale level) [4].
- precision and predictive gynecology (which implies the customizing, tailoring, and individualizing of healthcare provisions related to gynecology, based on the individual features of each patient).
- big and smart gynecology.
- 3D gynecology (which is the application of bioengineering and additive manufacturing processes to deposit bio-active materials, known as bio-inks, in order to develop and create tissue-like structures for regenerative or other medical purposes) [5].

Within this new conceptual framework [6], big data represents a massive set of data, characterized by different properties and features. These can be categorized both from a quantitative and qualitative standpoint, and include:

1. velocity (so-called “fast data”, which indicates the speed at which big data can be generated, released, and analyzed, almost in real-time, enabling data prediction, such as nowcasting or forecasting).
2. variety (because they can be produced by various channels and sources).
3. variability (since they are changeable and constantly in flux).
4. volume (related to the amount and magnitude of available data and information).
5. veracity/verification (related to data trustworthiness, accuracy, and reliability).
6. visualization (related to readability, affordability, and accessibility of data).
7. visibility (referring to the state of being able to be seen, this state is also known as “big visibility”).
8. virality (the speed at which data and related information spread along a people to people (P2P) network).
9. viscosity (or data friction or resistance, which refers to the heterogeneity of particularly complex data, which may be difficult to incorporate, integrate and combine).
10. virtual (referring to the digital/computational nature of big data).
11. value (in terms of advantages and benefits deriving from the collection, use, and analysis of big data). This implies that, after processing and analysis, big data can become smart and insightful Data.

Compared to other kinds of data, big data are difficult to store, pre-process, and analyze in an efficient way by utilizing classical techniques, thus requiring new computational resources, infrastructures, and ad hoc facilities.

Genomics, post-genomics (for instance, proteomics and metabonomics/metabolomics, among others) and biocomputational (bioinformatics or cheminformatics) repositories, clinical registries, imaging techniques and wearable sensors, and databases consulted by patients as well as by the lay people represent data streams producing molecular, clinical, imaging, and digital big data, respectively (as summarized in Table 1) [6,7].

## 2. The Aims of the Present Integrative Review

This comprehensive, integrative review aims at providing readers with an updated overview of the challenges and future research directions of big and smart data applied in the field of gynecology. The different types of big data will be presented, together with some of their major examples and applications for enhancing human female fertility, reproductivity, and treating gynecological diseases. Ethical implications will also be envisaged.

## 3. Gynecology and OMICS Data

Diseases result from the complex, multi-factorial interplay between a variety of exogenous and endogenous factors, including biological/individual, behavioral (lifestyles, such as smoking, dietary habits, and physical activity), and environmental variables. As such, they have unique molecular signatures, in terms of prognosis, response to pharmacological agents, co-morbidity, mortality, and survival rates. In this sense, the same disease may be considered as an umbrella term, collecting various pathophysiological and clinical manifestations. Deep phenotyping (quantitative histo-pathology/cyto-pathology) and clustering techniques enable us to differentiate and stratify patients in terms of outcomes and prognosis, such as response to drugs, surgery, or other treatment options, thus potentially guiding and informing the decision-making process according to available evidence [8,9].

Genomics studies can help identify a panel of genetic biomarkers, aimed at early detection of gynecological disorders (such as malignancies) and curb their morbidity/mortality rates. For instance, for ovarian cancer, a panel made up of cancer antigen 125 (CA-125), human epididymis protein 4 (HE4), cancer antigen 19-9 (CA 19-9), epidermal growth factor receptor (EGFR), granulocyte colony-stimulating factor (G-CSF), eotaxin, interleukin-2 receptor (IL-2R), circulating vascular cell adhesion molecule-1 (cVCAM), and macrophage migration inhibiting factor (MIF) seems to exhibit a satisfactory specificity and sensitivity [10]. The Gene Expression Predictor of High-grade Serous Ovarian Carcinoma Molecular SubTYPE (PrOTYPE) is another example of genomic panel comprising of 55 genes that has been developed and validated for predictive purposes in ovarian cancer patients, utilizing published gene expression data from 1650 cancers. Analyses have shown a statistically significant association between a set of variables, including age, residual disease, tumor stage, and tumor-infiltrating lymphocytes, as well as other relevant clinical-pathological outcomes and biological parameters, with the panel exhibiting an accuracy greater than 95% [11].

Similar panels can be devised at the proteomics level (see, for instance [12,13,14,15]). For example, Timms et al. [15] exploited different complementary profiling platforms and strategies to identify biomarker candidates in a statistically robust fashion. Authors were able to pin down four genes (namely, alpha-1 antitrypsin or A1AT, secretory leukocyte peptidase inhibitor or SLPI, apolipoprotein A-IV or APOA4, and vitamin D binding protein or VDBP) that could significantly differentiate between malignant and benign cases. On the other hand, either alone or even when combined, these biomarkers failed to overperform with respect to the “classical” biomarker CA-125, which remains the gold standard in terms of accuracy for diagnosing ovarian cancer. However, SLPI seemed to act as a satisfactory early biomarker, even though data were too preliminary and no firm conclusion could be drawn, thus warranting further research.

Furthermore, new, emerging biomarkers have been discovered in the fields of genomics and proteomics, like exosomes, which are involved in the cellular cross-talks and related biological processes [16]. Nutrigenomics [17,18], epigenomics [19] and nutriepigenomics [20,21] investigate how dietary habits and nutrition, as well as environmental factors, and their complex, non-linear interactions can finely modulate the human genome and its expression, and impact on human diseases, including gynecological conditions. For instance, a study explored the relationship between folic acid supplementation and one carbon metabolism gene single nucleotide polymorphisms (SNPs) on small-for-gestational-age (SGA) births [17]. Other studies assessed the association between pregnancy and childhood epigenetics [19,20].

Besides proteomics, other post-genomics specialties include transcriptomics [22] and cytomics [22,23,24], which enable the study of gene transcripts and biochemistry and biophysics at the single cellular level, respectively. Another emerging OMICS discipline aims at exploring and dissecting the molecular basis of the vaginal microbiome, conceived as a specific compartment of the human microbiome [25]. The vagina is characterized by specific features, such as the presence of few bacteria, in contrast to other parts of the human body. These microbes include glycogen-using, lactic acid-producing, estrogen-dependent *lactobacilli*, and other fermentative microbial species, which, together with epithelial cells, are responsible for the acid vaginal pH [26].

Various vaginotypes exist based on the composition of vaginal microbiota, which can have an impact on vaginal dysbiosis, vaginosis, sexually transmitted diseases (STDs), such as the infections by human papillomavirus (HPV), and birth and pregnancy outcomes [27,28,29,30,31,32,33]. As such, ‘vaginal and maternal microbiomics’ is an emerging field of super-specialties within microbiomics, which appears promising in terms of predictive power. This paves the way for tailored therapeutics—the so-called pharmacomicrobiomics and pharmacoculturomics [34], that is to say, drugs designed and developed based on our knowledge of molecular interactions between the human vaginal microbiome and potential pharmacological agents.

Metabolomics/metabonomics [35], exosome metabolomics (exometabolomics) [35], and multi-OMICS approaches [36,37] represent further innovative strategies for (sub-)typing gynecological patients. Finally, phenomics [38] aims at systematically and comprehensively measuring and quantitatively assessing the human phenome, which is the array of physical, chemical, biochemical, and biophysical traits expressed and/or generated by an individual during its development, adaptation to genetic influences, and environmental stimuli. For instance, in Denmark, Westergaard et al. [38] carried out a phenome-wide study to explore the molecular basis of recurrent pregnancy loss (RPL) utilizing a cohort of 1,370,896 never-pregnant women aged 12–40 years in the study period between 1977 and 2016. The authors used a population-wide healthcare registry and found that RPL represented a major risk factor for a wide array of disorders, including cardiovascular, autoimmune, and mental health diseases. This could shed light on the etiopathogenetic mechanisms underlying RPL and provide new management and treatment options, warranting further research in the field. Other phenomics studies have explored gynecological disorders, such as polycystic ovary syndrome [39] and reproductive issues [40,41,42,43,44].

All these OMICS disciplines and specialties are briefly overviewed in Table 2.

## 4. Gynecology and Computational/Clinical Data

There exist several high-quality databases in the field of gynecology. For instance, the PREgnancy Care Integrating translational Science, Everywhere (PRECISE) database [45] is a unique resource for gynecologists and scholars working in the field of female reproductive health. This registry represents a core infrastructure of a data collection platform that has been developed and devised based on outcome data harmonization, and data recording and reporting standardization, relying on open-access technologies and clinical collaborating networks. It combines socio-demographic and clinical information with biological/biomedical parameters, including Human Immunodeficiency Virus (HIV) status, tests performed (including blood pressure measurement), therapeutical management received (for instance, iron therapy or uterotonic usage after delivery), postpartum maternal assessment within 48 h of birth, and newborn resuscitation, immediate skin-to-skin contact, and immediate drying, among others.

Other databases relevant to gynecology include: the Center of Excellence in Minimally Invasive Gynecology (COEMIG) registry, the Patient Centered Results for Uterine Fibroids (COMPARE-UF) database, the National Quality Registry for Gynaecological Surgery (GynOp) registry, the PanCare Studies in Fertility and Ototoxicity to Improve Quality of Life after Cancer during Childhood, Adolescence and Young Adulthood (PanCareLIFE) database, the Pelvic Floor Disorders (PFD) registry and the Society for Assisted Reproductive Technology (SART) registry.

Registries can also include patient-reported outcome measures (PROMs), thus providing fundamental information about a patient’s perspective on gynecological management in terms of adherence and compliance to treatment and therapeutical preferences [46]. As such, they represent valuable resources for physicians and gynecologists. Some of the major big data-based databases available in the field are summarized in Table 3.

## 5. Gynecology and Imaging/Wearable Sensor Data

A new big data-based specialty, termed radiomics, is emerging, which exploits radiographic medical images utilizing artificial intelligence-based algorithms, such as data-characterization or automatic segmentation and interpretation techniques, to uncover hidden trends and patterns and extract mineable information [47] in order to predict, for instance, the prognosis of patients suffering from malignancies [48]. For instance, Rizzo et al. [48] carried out a systematic review of the literature to evaluate the predictive power of radiomics in properly estimating overall survival and progression free survival in ovarian cancer patients. Based on six articles, statistically significant associations between radiomic features and survival rates could be found.

An unprecedented wealth of data can also be obtained by using wearable sensors and related technologies [49], which can collect information concerning women’s periods, ovulation cycles, and breastfeeding habits, as well as other bodily functions. As such, IoT and IoMT can really advance gender equality and empower women. For instance, Runkle et al. [50] investigated the use of such sensors during prenatal care. Authors conducted a web questionnaire-based study, utilizing a 21-item survey, which was delivered to 28 obstetrics and gynecology or family medicine providers. Another 21-item paper survey was administered to a sample of 103 pregnant women. Authors were able to find high acceptability levels of mobile and digital technologies both among healthcare providers and patients. Noteworthy, approximately 70% of women were in favor of utilizing smartphones for receiving individualized suggestions during their pregnancy, expressing their willingness to change their behaviors and adopt healthier lifestyles. Women were also interested in health and environmental tracking and monitoring. Similarly, healthcare providers expressed interest in mobile and smart technologies being used in tandem with ‘classical’ ones, stating that they could help them in their daily medical practice and feeling that they would have an increasing role in tele-gynecology.

In another study, Niela-Vilén et al. [51] were able to assess pregnant women’s well-being during the ongoing coronavirus (COVID-19) pandemic by means of smartwatch technology. Authors performed a longitudinal cohort study recruiting two cohorts of women: one during the first COVID-19 wave with a history of preterm births or late miscarriages, and one during the second COVID-19 wave without pregnancy loss and with a history of full-term births. Physical activity practice significantly decreased, due to COVID-19 induced public health measures (such as quarantine, lockdown, and social distancing) and pregnancy, whereas sleep time was impacted by pregnancy but not by public health restrictions. Heart rate variability changed as well with respect to baseline values, with stress levels being significantly increased.

## 6. Gynecology and Infodemiology

‘Infodemiology’ (a portmanteau of ‘information’ and ‘epidemiology’) and ‘infoveillance’ (a combination of the words ‘information’ and ‘surveillance’) are the novel, emerging sciences of “distribution and determinants of information in an electronic medium, specifically the internet, or in a population, with the ultimate aim to inform and improve public health and public policy” [52].

Despite the increasing importance of the web in contemporary society and its usage by patients [53,54], in the existing scholarly literature, very few studies have addressed such a topic. For instance, Rezniczek et al. [55] retrieved and quantitatively assessed a set of 672 websites related to gynecological topics from several countries, including Switzerland (n = 49), Austria (n = 57), and Germany (n = 566). Websites were objectively ranked utilizing a composite panel of criteria: namely, the 2-item Google search ranking, a detailed, ad hoc devised checklist regarding technical aspects (in total, 11 items), web navigation and surfing (in total, 8 items), and website content (in total, 6 items) for an overall 26-point score. Scores were compared nationally (within a country) and internationally (between countries). The average score of websites was computed to be 13.8 ± 3.3, with only 4.2% of websites being rated as good. Most online resources (61.8%) were judged to be fair. Professional affiliation (academic versus non-academic), size and dimension of the institution (single versus consortium), and geographical location were found to be the determinants of the overall score. At the multivariate regression analysis, all sub-scores (Google search ranking, technical aspects, web navigation and surfing, and website content) could statistically predict the overall score, with the latter sub-score being the most significant one. Finally, all sub-scores exhibited statistically robust correlations among each other.

Han et al. [56] carried out a cross-sectional study of internet websites related to abortion, in order to investigate its relationship with psychological wellbeing and safety, depression, and mental health, as well as infertility. For this purpose, they exploited the Google AdWords tool to retrieve the keywords most associated with the topics under study and Google Trends to monitor and track Google’s search engine to build a list of databases of websites commonly consulted. These sites were then categorized and assessed in terms of content and attitude towards abortion, reliability and accuracy, and type of online resource. Authors collected and analyzed 316 websites: more than half of them (57.3%) were unbiased and neutral towards abortion, with approximately one third (35.4%) being clearly anti-choice, and only 7.3% being pro-choice. The average trustworthiness score varied among websites according to their content and attitude, being higher in neutral and pro-choice sites, with respect to anti-choice ones.

Endometriosis is a non-lethal but highly disabling gynecological condition characterized by infertility, pelvic pain and fatigue, allergies, and gastro-enterological problems. Hirsch et al. [57] mined five popular internet search engines (namely, aol.com, ask.com, bing.com, google.com, and yahoo.com), searching for endometriosis-related web resources. The authors were able to identify 54 relevant websites out of an initial list of 750 websites. These digital resources were assessed in terms of content credibility, quality, readability, and accuracy. The authors found that over a third and approximately half of these websites failed to attribute authorship and report the sources of information or to back up the online material with scholarly references, respectively. Noteworthy, authors could not identify any website disseminating endometriosis-related information written in plain English, with very few websites being judged as high-quality resources and with only one website displaying reliable, evidence-based information (namely, evidentlycochrane.net). Generally speaking, websites focused on diagnosis of endometriosis, without discussing other topics relevant for patients suffering from this gynecological disorder and mentioning content limitations. In conclusion, no website succeeded in achieving a high score in all four domains assessed by the authors (credibility, quality, readability, and accuracy).

Adawi et al. [58] conducted an infodemiological study in order to assess endometriosis-related digital activities in terms of web searches, after that an Italian celebrity, the television presenter Rossella Brescia, publicly announced that she was affected by this gynecological issue. More specifically, authors carried out a retrospective search of digital seeking behavior related to endometriosis in Italy, over the previous 5 years, exploiting Google Trends (section Google News) and utilizing “endometriosis” as the keyword and “search topic” as the search strategy option. The impact of media coverage and news dissemination on web behavior was also explored. The authors were able to find a statistically borderline significant association between Rossella Brescia and endometriosis related web searches. The self-disclosure of the celebrity impacted digital behavior, resulting in bursts of web activity.

In conclusion, based on this literature overview, we can state that physicians, and in particular gynecologists, should become aware of the increasing role of new media and social networks in contemporary society. Patients surf the web to retrieve information about their condition and share details with other patients, creating online communities [59,60]. However, patients have a higher chance of encountering websites spreading biased, inaccurate, and misleading material. As such, doctors should significantly strengthen their presence online and make better use of this means of interaction with patients [60,61]. Physicians’ communication, while preserving trustworthiness and scholarly rigor, should be accessible to the lay public, aiming at improving their health literacy, promoting healthy lifestyles, and increasing and enhancing the awareness of preventative strategies. To this end, advocacy and the celebrity effect could be exploited [58,62].

## 7. An Overview of Use of Big Data in the Field of Gynecology

In summary, big data has so far been exploited in different sub-fields of gynecology, ranging from human reproductive health, physiology, and physiopathology (including pregnancy, infertility, and abortion) to assisted reproduction, sexually transmitted diseases (e.g., HPV), reproductive disorders (like endometriosis), and malignancies (endometrial, cervical, and ovarian cancers). Big data has enabled the collection of insightful information concerning early disease detection, stratification, and prognosis, as well as about PROMs.

These are briefly summarized in Table 4.

## 8. Pitfalls and Limitations of Big Data

Despite its strengths, big data is not immune from shortcomings and drawbacks [63]. These can be both technical and ethical/legal/bio-ethical. Among technical challenges, data portability refers to the possibility of transferring data from a given device, and format to other devices and formats, preserving their readability. Another particular technical challenge of Big Data is, given their variety and variability, to combine and organize them within a unique, coherent framework that can really advance our understanding of female reproductive health and gynecological disorders. This fragmentation and heterogeneity of data sources and types may challenge the classical concepts of ethical consent, data access, data sharing, and the distinction between data owner/legal custodian/buyer and data user (for example, researcher, stakeholder, citizen, etc.)—which is becoming more and more blurred. Data privacy (i.e., handling data in such a way as to minimize the risk of data theft/breach and to ensure the protection of sensitive information), data protection especially for unstructured data (personally identifiable), data integrity, data transparency, data replicability/reproducibility and other (bio-)ethical concerns represent further pitfalls of big data. In more detail, these include risk assessment and risk analysis, and uncertainty of communication to lay people, among others [64]. These are briefly summarized in Table 5.

Specifically concerning gynecology, and regarding human reproductive health in general, information related to a patient’s health status is particularly sensitive. Pregnancy is also a period in which women could experience privacy concerns. However, some studies [50] have demonstrated high levels of acceptability among patients towards big data and computational/digital technologies (such as mobile sensors), with pregnant women being comfortable with utilizing such devices and sharing information from these tools with their physicians [50].

However, few data concerning these attitudes are available and there is a gap in the literature concerning this specific topic. Future high-quality studies should be conducted examining the determinants of acceptability of big data based smart technologies among women.

## 9. Areas for Future Research

While some big data-based resources, such as large, nation-wide epidemiological surveys and claim databases, are already available for gynecologists, they are not adequately trained to exploit such tools. Medical students, residents, and future practitioners should be instructed and educated about the opportunities offered by big and smart data. This is of paramount importance, in that data mining can potentially result in a high number of parameters being statistically significant and gynecologists should assist in the process of decision-making and critical and meaningful interpretation of results, being able to distinguish between statistical and clinical significance. Involving practitioners in translational research could facilitate and expedite the transition from bench to bedside, helping to fill in several gaps of knowledge, conjugating, integrating and harmonizing discovery bioscience and clinical care.

## 10. Conclusions

Within the new theoretical framework of the ‘disruptive innovation medical era’, big and smart data are anticipated to revolutionize different medical specializations, including gynecology, shedding light on female reproductive health, both in terms of physiology and pathophysiology. More specifically, big data appears to be promising, in the field of gynecology, to potentially increase its accuracy and precision, stratify patients, provide opportunities for personalized treatment options rather than delivering a package of “one-size-fits-it-all” healthcare management provisions, and enhance its effectiveness at each stage (health promotion, prevention, diagnosis, prognosis, and therapeutics). However, research to overcome the technical and ethical limitations and pitfalls of big data is warranted.

## Figures and Tables

**Table 1:** An overview of the different kinds of Big Data.

Type of Big Data	Sources
Molecular Big Data	Wet-lab, microarrays Bioinformatics/cheminformatics repositories
Computational/Clinical Big Data	Electronic Health Records (EHRs) and clinical databases
Imaging Big Data	Wearable sensors, imaging approaches
Digital Big Data	Website searches

**Table 2:** An overview of the main OMICS disciplines useful for gynecologists and scholars working in the field of human female reproductive health: bridging the gaps from genomics to phenomics, combining/merging the various specialties via multi-OMICS integration.

OMICS Discipline	Example	References
Genomics	Cancer genomics	[10,11]
	Nutrigenomics	[17,18]
	Epigenomics	[19]
	Nutriepigenomics	[20,21]
	Exosome genomics	[16]
Proteomics	Cancer proteomics	[12,13,14,15]
	Exosome proteomics	[16]
Transcriptomics	Cancer transcriptomics	[22]
Cytomics	Cancer cytomics	[22,23,24]
Metabolomics/metabonomics	Metabolomics	[16,35]
	Exometabolomics/microbial exometabolomics	
Microbiomics	Vaginal and maternal microbiomics; microbial culturomics	[25,26,27,28,29,30,31,32,33]
Pharmacomicrobiomics and pharmacoculturomics	Cancer pharmacomicrobiomics	[34]
Multi-omics	Multi-omics of preterm birth	[36]
	Multi-omics of gynecological cancers	[37]
Phenomics	Cancer and infertility phenomics	[38,39,40,41,42,43,44]

**Table 3:** An overview of major big data-based databases useful for gynecologists and scholars working in the field of human female reproductive health.

Database	Extended Title
COEMIG	Center of Excellence in Minimally Invasive Gynecology
COMPARE-UF	Patient Centered Results for Uterine Fibroids
GynOp	National Quality Registry for Gynaecological Surgery
PanCareLIFE	PanCare Studies in Fertility and Ototoxicity to Improve Quality of Life after Cancer during Childhood, Adolescence and Young Adulthood
PFD Registry	Pelvic Floor Disorders
PRECISE Registry	PREgnancy Care Integrating translational Science, Everywhere
SART Registry	Society for Assisted Reproductive Technology

**Table 4:** Aspects and sub-fields of gynecology that can benefit from the use of big data.

Gynecology Sub-Field Potentially Interested by Big Data	Examples
Reproductive health (pregnancy, infertility, abortion, endometriosis) and link to mental health and psychological well-being	Physiological and physio-pathological insights Health-related literacy
Assisted reproduction	Public interest and health-related literacy
Sexually transmitted diseases (such as HPV)	Health-related literacy
Gynecological cancers (endometrial, cervical, and ovarian cancers)	Molecular and cellular characterizations Clinical outcomes Health-related literacy

**Table 5:** The main pitfalls and limitations plaguing the usage of big data.

Potential Pitfall
***Technical Challenge***
Data integration and combination
Data portability
***Ethical/Legal Challenge***
Blurred distinction between data owner and data user
Ethical consent
Data privacy and data protection
Data portability
Data sharing
Data integrity
Data transparency
Data replicability/reproducibility

## Data Availability

Not applicable.
